# Optimizing Available Phosphorus in Calcareous Soils Fertilized with Diammonium Phosphate and Phosphoric Acid Using Freundlich Adsorption Isotherm

**DOI:** 10.1155/2013/680257

**Published:** 2013-11-06

**Authors:** Asif Naeem, Muhammad Akhtar, Waqar Ahmad

**Affiliations:** ^1^Soil Science and Plant Nutrition, School of Earth and Environment, University of Western Australia, Crawley, WA 6009, Australia; ^2^Nuclear Institute for Agriculture and Biology, P.O. Box 128, Jhang Road, Faisalabad 38000, Pakistan; ^3^Institute of Soil and Environmental Sciences, University of Agriculture, Faisalabad 38040, Pakistan

## Abstract

In calcareous soils, phosphorus (P) retention and immobilization take place due to precipitation and adsorption. Since soil pH is considered a major soil variable affecting the P sorption, an acidic P fertilizer could result in low P adsorption compared to alkaline one. Therefore, P adsorption from DAP and phosphoric acid (PA) required to produce desired soil solution P concentration was estimated using Freundlich sorption isotherms. Two soils from Faisalabad and T. T. Singh districts were spiked with 0, 10, and 20 % CaCO_3_ for 15 days. Freundlich adsorption isotherms (*P* = *aC* ^*b*/*a*^) were constructed, and theoretical doses of PA and DAP to develop a desired soil solution P level (i.e., 0.20 mg L^−1^) were calculated. It was observed that P adsorption in soil increased with CaCO_3_. Moreover, at all the levels of CaCO_3_, P adsorption from PA was lower compared to that from DAP in both the soils. Consequently, lesser quantity of PA was required to produce desired solution P, 0.2 mg L^−1^, compared to DAP. However, extrapolating the developed relationship between soil CaCO_3_ contents and quantity of fertilizer to other similar textured soils needs confirmation.

## 1. Introduction

In calcareous soils, phosphorus (P) retention and mobilization take place due to precipitation and adsorption; however, it is not always easy to distinguish between the two mechanisms. Water soluble P fertilizers applied to soil react with the soil constituents to form less soluble phosphates. When added to soil containing large amounts of calcium, soluble P is usually precipitated as dicalcium phosphate or octacalcium phosphate [1]. At low P concentration up to 0.4 mg L^−1^, active CaCO_3_ and/or Fe dithionite could result in P adsorption whereas, at high concentration, precipitation could be predominant over the adsorption process [2]. The reactivity of CaCO_3_ in soils depends upon the specific surface area of the carbonate and on its total surface area [3]. It has been demonstrated [4] that Ca^2+^ is dominant ion in soil solution of calcareous soils and it is possible that formation of less soluble complexes with weak acid anions like orthophosphate is due to unavoidable dominance of this ion. The dynamics of P is managed by calcite, which strongly holds P and consequently maintains low P concentration in soil solution. It was noted [2] that low CaCO_3_ showed an upper limit of P adsorption varying from 1.4 to 3.5 mg P kg^−1^ that was not modified by further increment of P in solution. Conversely, in soil with high CaCO_3_ content, P adsorption increased up to the maximum experimental concentration of P in solution (2 g L^−1^).

As the soil pH is considered a major soil variable affecting the P sorption, thus an acidic P fertilizer could result in low P adsorption compared to basic one resulting in less amount of fertilizer required to produce P concentration in soil solution optimum for plant growth. Amount of P required to bring its desired concentration in soil solution could be better determined by P sorption isotherms [5, 6] instead of conventional soil P test; those do not take into consideration the physicochemical properties of soil. Although both the Freundlich and Langmuir isotherms describe the adsorption phenomena satisfactorily [7], the former is preferred because it is capable of rigorous derivation and correlates well with soil properties [8]. Moreover, it is based on assumptions more realistic than some other cases; that is, an adsorption maximum is not obtainable from the isotherm that seems compatible with most of the observed P sorption by soils, at least, under normal laboratory conditions. Keeping in view the above facts, a laboratory study was conducted using Freundlich adsorption isotherm to assess the P adsorption capacity of two soils when treated with PA and DAP at varying levels of CaCO_3_.

## 2. Materials and Methods

### 2.1. Soil Preparation and Analyses

Surface soil samples were collected from Faisalabad and T. T. Singh districts (hereafter referred to as S-I and S-II, resp.), air-dried, passed through a 2 mm sieve, mixed thoroughly, and stored in labeled plastic bottles. Samples were analyzed for various physiochemical properties like texture [9], pH of saturated paste (pH_s_), electrical conductivity of saturation extract (EC_e_) [10], available K [11], Olsen P [12], organic matter [13], and calcium carbonate [14].

### 2.2. Development of CaCO_3_ Levels in Soils

One kg of each soil was taken in plastic buckets, and three levels of CaCO_3_ (native, 10%, and 20%) were developed by mixing reagent grade salts with soils. The soils were wetted with distilled water to attain field capacity and equilibrated for 15 days at room temperature. At the termination of incubation, soils were mixed, dried, and passed through a 2 mm sieve and stored in plastic bottles for use in adsorption studies.

### 2.3. Adsorption Isotherms

Adsorption isotherms were constructed using a series of solutions with P concentrations (2.5, 5, 7.5, 10, 20, 40, and 80 ppm) prepared from each of DAP and PA in 0.01 M CaCl_2_. To 2.5 g samples of the soils, 25 mL of the above-said P solutions was added and shaken for 24 h on a mechanical shaker. After equilibration, the samples were centrifuged for 15 min. at 4000 rpm and filtered through Whatman number 42 filter paper. Phosphorus concentration in the final solutions was determined following the method of Murphy and Riley [15]. The difference in P concentration of solutions before and after equilibrium was taken as the amount of P adsorbed. The sorption isotherms were examined by modified Freundlich equation proposed by Le Mare [16] as follows:

(1) $$\begin{array}{c} P=aC^{b/a}, \end{array}$$

where *P* is amount of P adsorbed per unit of soil (*μ*g g^−1^), *C*  is  equilibrium P concentration in soil solution (*μ*g mL^−1^), and *a* and *b* are the amount of P adsorbed and the buffer capacities, respectively. The parameters *a* and *b* were estimated by regression of the logarithmic form of the data obtained from adsorption isotherms. Theoretical doses of PA and DAP fertilizers to develop a desired soil solution P level, that is, 0.20 mg L^−1^, were calculated. A regression between calculated quantities of P fertilizer and CaCO_3_ levels was developed to estimate requirement of P fertilizer for any level of soil CaCO_3_. 

## 3. Results and Discussion

### 3.1. Freundlich Adsorption Isotherms for CaCO_3_ Amended Soils

**Figure 1:**
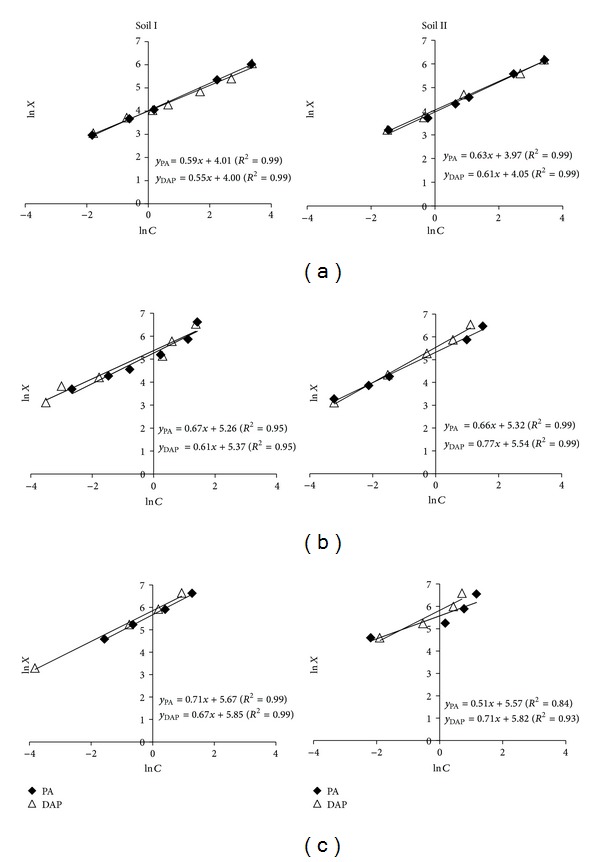
Freundlich isotherms for P adsorption: (a), (b), and (c) represent native, 10%, and 20% CaCO_3_, respectively.

The physical and chemical properties of the soils are presented in Table 1. Both the soils were nonsaline, silty clay loam in texture, and slightly alkaline in reaction. After constructing the P adsorption isotherms, the data were subjected to examine the fitness of modified Freundlich equation. Linear plot of the modified Freundlich equation presented in Figure 1 and parameters of the equation (amount adsorbed (*a*), buffer capacity (*b*) mL g^−1^, and correlation coefficient (*r* ^2^)) are presented in Table 2. The goodness of the fit of the model was ascertained from *r* ^2^ values (≥0.84) which indicated high conformity of the adsorption data with the Freundlich model. These findings are in agreement with those of Chaudhry et al. [17] and Sarfraz et al. [18] who also reported dependence of the exponent of Freundlich equation on solution P concentration instead of time and temperature. A good fit of the P adsorption data to the Freundlich adsorption model over the Langmuir and Tempkin was also reported by Khan et al. [19].

### 3.2. Calcium Carbonate and P Adsorption

In adsorption equation, *b* represents the buffer power of the soil for P. The more the value of *b* is the more the P adsorption capacity of soil would be. The soils differed slightly in buffer capacities despite a large difference in native CaCO_3_ that might be due to similar proportion of active CaCO_3_ and its specific surface area in the soils which mainly govern P behavior. With the addition of CaCO_3_ in soils, the buffer capacity of the soils was increased (Table 3). Similarly, Samadi and Gilkes [20] and Samadi [21] reported that P adsorption in calcareous soil was related to CaCO_3_ contents. Castro and Torrent [22] found an increase in differences among P fertilizers for P adsorption with the increase in carbonate contents of the soil and attributed the fact to the precipitation of Ca-phosphate. However, Samadi [23] reported that both total and active CaCO_3_ were less important factors for P adsorption. This discrepancy in results has been answered by Peña and Torrent [24] as the inability of the standard methods used for the determination of total and active CaCO_3_. 

### 3.3. Phosphorus Requirement as a Function P Source

It was observed that, at all the levels of CaCO_3_, P adsorption from PA was lower compared to that from DAP in both the soils. Consequently, lesser quantity of PA was required to produce desired solution P, 0.2 mg L^−1^, compared to DAP (Table 4). Lower P adsorption and/or precipitation from PA compared to DAP might be due to higher acidity produced by PA in alkaline soil. Although there is limited information available comparing the effect of acidic and alkaline P source on its adsorption/precipitation in soil, our results are in line with the information available so far. According to Lu et al. [25], SSP being an acidic P fertilizer performed better than DAP for P uptake and soil test levels on alkaline calcareous soil. Similarly, in a two-year field experiment Chaubey and Kaushik [26] reported higher grain yield of wheat with SSP compared to DAP and attributed the low yield with DAP to more P fixation as a result of alkaline soil pH around its granule. Wijewardena [27] observed that the highest available P content in soils after potato and vegetables harvest soils for consecutive four seasons with TSP compared to imported and local Siri Lankan rock phosphates was lesser in acidity. 

### 3.4. Phosphorus Requirement as a Function of CaCO_3_

**Figure 2:**
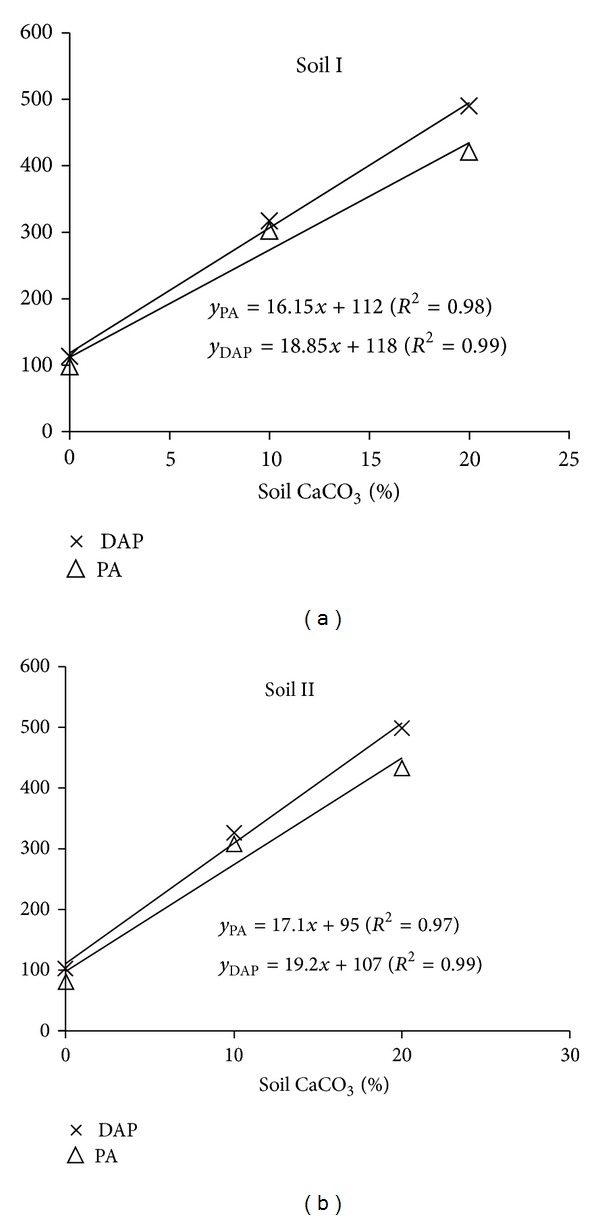
Relationship between soil CaCO_3_ and P_2_O_5_ requirement (kg ha^−1^) to achieve 0.2 mg L^−1  ^soil solution P.

Regression between soil CaCO_3_ and solution P is presented in Figure 2 for both soils and P sources. Using these equations, the amount of P fertilizer required for any level of CaCO_3_ could be calculated. Use of Freundlich P sorption isotherm, which relates soil solution P concentration with quantity of P adsorbed in soil, to predict P fertilizer requirement of a specific soil is better approach rather than using soil test. It may be due to that soil test only provides information about the plant available P [28] and does not estimate the amount of fertilizer P needed unless calibrated for a particular test. But extrapolating the developed relationship between soil CaCO_3_ contents and quantity of fertilizer to other similar textured soils needs confirmation. If it holds true, then it would be quite promising, time saving, and accurate approach for predicting P fertilizer requirement to achieve the desired level of soil solution P.

## 4. Conclusion

In semiarid regions, CaCO_3_ is the dominant soil constituent limiting P availability to plants by adsorption and precipitation reactions. Therefore, P addition to such soil could be rationalized depending upon CaCO_3_ contents of soil. A good fit of the adsorption data to the modified Freundlich model in the present study suggests that external P requirement of plants could be better determined using this adsorption model rather than using soil test P values depicting available phosphorus. Moreover, using an acidic P source instead of alkaline one could result in lesser P adsorption and/or fixation in alkaline calcareous soils. 

## Figures and Tables

**Table 1:** Physiochemical properties of the soils used in adsorption studies.

Properties	Unit	Value	
		S-I	S-II
pH_s_	—	7.20	7.30
EC_e_	dS m^−1^	1.40	3.86
CaCO_3_	%	6.58	14.06
Organic matter	%	1.18	1.13
Olsen P	mg kg^−1^ soil	15.56	9.35
Sand	%	15.53	20.12
Silt	%	47.45	47.54
Clay	%	37.53	32.52
Textural class	—	Silty clay loam	Silty clay loam

**Table 2:** Fitted Freundlich adsorption isotherms.

CaCO_3_ level (%)	Phosphoric acid treated soils		DAP treated soils	
	S-I	S-II	S-I	S-II
Native	*P* = 55.37*C* ^0.59^	*P* = 52.87*C* ^0.63^	*P* = 54.64*C* ^0.55^	*P* = 57.51*C* ^0.61^
10%	*P* = 192.87*C* ^0.64^	*P* = 216.15*C* ^0.68^	*P* = 215.29*C* ^0.61^	*P* = 242.52*C* ^0.77^
20%	*P* = 289.88*C* ^0.71^	*P* = 267.04*C* ^0.50^	*P* = 348.06*C* ^0.68^	*P* = 335.82*C* ^0.71^

**Table 3:** Buffer capacities of CaCO_3_ enriched soils as determined from Freundlich adsorption isotherms.

CaCO_3_ level (%)	PA		DAP	
	S-I	S-II	S-I	S-II
Native	33 ± 1.63*	33 ± 1.98	30 ± 1.72	35 ± 2.07
10%	128 ± 5.57	121 ± 3.40	145 ± 4.50	175 ± 8.89
20%	206 ± 11.95	211 ± 9.54	239 ± 9.01	248 ± 15.41

*Means ± SE.

**Table 4:** Quantities of P_2_O_5_ (kg ha^−1^) required to achieve 0.2 mg L^−1^ soil solution P.

CaCO_3_ level (%)	PA		DAP	
	S-I	S-II	S-I	S-II
Native	98 ± 6.29*	78 ± 5.69	113 ± 7.94	99 ± 5.89
10%	302 ± 8.67	298 ± 12.49	317 ± 14.42	316 ± 9.50
20%	421 ± 14.65	419 ± 8.60	490 ± 20.13	483 ± 12.98

*Means ± SE.

## References

[1] Bell LC, Black CA (1970). Transformation of dibasic calcium phosphate dihydrate and octacalcium phosphate in slightly acid and alkaline soils. *Soil Science Society of America Journal*.

[2] Papini R, Castelli F, Panichi A (1999). Phosphorus retention and leaching in some sandy soils of Northern Italy. *Italian Journal of Agronomy*.

[3] Rashid A, Rowell DL (1988). Phosphate sorption and release: 1. Isotopically exchangeable and non-exchangeable adsorbed phosphate in relation to soil properties. *Pakistan Journal of Soil Science*.

[4] Bertrand I, Hinsinger P, Jaillard B, Arvieu JC (1999). Dynamics of phosphorus in the rhizosphere of maize and rape grown on synthetic, phosphated calcite and goethite. *Plant and Soil*.

[5] Sposito G (1980). Derivation of the Fremdlich equation for ion exchange reactions in soils. *Soil Science Society of America Journal*.

[6] Holford ICR (1997). Soil phosphorus: its measurement, and its uptake by plants. *Australian Journal of Soil Research*.

[7] Boschetti ANG, Quintero GCE, Benavidez QRA (1998). Characterization of the capacity factor of phosphorus in soils of Entre Rios, Argentina. *Brasileira-De-Ciencia-Do-Solo*.

[8] Hussain A, Ghafoor A, Haq MA, Nawaz M (2003). Application of the Langmuir and Freundlich equations for P adsorption phenomenon in saline-sodic soils. *International Journal of Agriculture and Biology*.

[9] Bouyoucos GJ (1962). Hydrometer method improved for making particle size analysis of soils. *Agronomy Journal*.

[10] US Salinity Lab. Staff (1954). *Diagonsis and Improvement of Saline and Alkali Soils*.

[11] Helmke PA, Sparks DL, Sparks DL (1996). Lithium, sodium, potassium, rubidium, and cesium. *Methods of Soil Analysis. Part 3. Chemical Methods*.

[12] Olsen SR, Cole CV, Watanabe FS, Dean LA (1954). *Estimation of Available Phosphorus in Soils By Extraction with Sodium Bicarbonate*.

[13] Nelson DW, Sommers LE, Page AL (1982). Total carbon, organic carbon, and organic matter. *Methods of Soil Analysis*.

[14] FAO (1974). *The Euphrates Pilot Irrigation Project. Methods of Soil Analysis*.

[15] Murphy J, Riley JP (1962). A modified single solution method for the determination of phosphate in natural waters. *Analytica Chimica Acta*.

[16] Le Mare PH (1982). Sorption of isotopically exchangeable and non-exchangeable phosphate by some soils of Colombia and Brazil, and comparisons with soils of southern Nigeria. *Journal of Soil Science*.

[17] Chaudhry EH, Ranjha AM, Gill MA, Mehdi SM (2003). Phosphorus requirement of maize in relation to soil characteristics. *International Journal of Agriculture and Biology*.

[18] Sarfraz M, Abid M, Mehdi SM (2009). External and internal phosphorus requirements of wheat in Rasulpur soil series of Pakistan. *Soil & Environment*.

[19] Khan MA, Kim S-W, Rao RAK (2010). Adsorption studies of Dichloromethane on some commercially available GACs: effect of kinetics, thermodynamics and competitive ions. *Journal of Hazardous Materials*.

[20] Samadi A, Gilkes RJ (1999). Phosphorus transformations and their relationships with calcareous soil properties of southern Western Australia. *Soil Science Society of America Journal*.

[21] Samadi A Changes in added available phosphorus with time in contrasting calcareous soils with a mediterranean type of climate.

[22] Castro B, Torrent J (1994). Phosphate availability in calcareous Vertisols and Inceptisols in relation to fertilizer type and soil properties. *Fertilizer Research*.

[23] Samadi A (2006). Phosphorus sorption characteristics in relation to soil properties in some calcareous soils of Western Azarbaijan province. *Journal of Agricultural Science and Technology*.

[24] Peña F, Torrent J (1990). Predicting phosphate sorption in soils of mediterranean regions. *Fertilizer Research*.

[25] Lu DQ, Chien SH, Henao J, Sompongse D (1987). Evaluation of short-term efficiency of diammonium phosphate versus urea plus single superphosphate on a calcareous soil. *Agronomy Journal*.

[26] Chaubey AK, Kaushik MK (2000). Influence of levels and sources of phosphorus on yield and nodules dry weight of summer green gram. *The Madras Agricultural Journal*.

[27] Wijewardena JDH (1998). Effect of phosphorus sources and levels with particular emphasis on selectively mined Eppawela rock phosphate on vegetable production. *Journal of the National Science Council of Sri Lanka*.

[28] Fixen PE, Grove JH (1990). *Testing Soil for Phosphorus*.

